# Falls in oxygen saturations accompany electrographic seizures in term neonates: an observational study

**DOI:** 10.1038/s41390-024-03063-0

**Published:** 2024-02-16

**Authors:** David Wertheim, Anup C. Kage, Ivone Lancoma-Malcolm, Caroline Francia, Michael Yoong, Divyen K. Shah

**Affiliations:** 1 Faculty of Engineering, Computing and the Environment, Kingston University, Surrey, UK; 2 Neonatal Intensive Care Unit, Royal London Hospital, Barts Health NHS Trust, London, UK; 3 Barts and the London School of Medicine and Dentistry, Queen Mary University of London, London, UK; 4 Department of Paediatric Neurology, Royal London Hospital, Barts Health NHS Trust, London, UK

## Abstract

**Background:**

Effective seizure detection is important however, clinical signs of seizure activity may be subtle in neonates. This study aimed to systematically investigate SpO_2_ and respiratory pattern changes associated with EEG seizures in term-born neonates.

**Method:**

An observational study in term neonates at risk of seizures admitted to a single tertiary level neonatal intensive care unit. Synchronised high-resolution physiological data (ECG, pulse oximetry, respiration) and EEG/amplitude-integrated EEG (aEEG) monitoring were recorded. Sections of traces with evidence of clear EEG seizure activity were compared with physiological data recorded at the same time.

**Results:**

22/44 (50%) neonates who had aEEG monitoring were noted to have electrographic seizures. Physiologic download measurements were available for 11 of these neonates. In nine of these, an acute drop in oxygen saturation (SpO_2_) of at least 5% was noted in at least one seizure. Accompanying apnoeas were noted in three neonates.

**Conclusion:**

Acute decreases in SpO_2_ were seen in term neonates associated with seizures and these were not always accompanied by an apnoeic episode. Physiologic download in association with EEG monitoring may assist in improving seizure detection. Unexplained drops in SpO_2_ could indicate further investigation for possible seizures in at-risk neonates.

**Impact:**

- A decrease in blood oxygen saturation (SpO_2_) associated with EEG seizures can occur in term infants with HIE or perinatal stroke.
- Drops in SpO_2_ associated with EEG seizures in term infants with HIE or stroke may occur in the absence of apnoeas.
- Unexplained acute falls in SpO_2_ in sick neonates may suggest possible seizures.
- Drops in SpO_2_ associated with seizures in term infants can occur over less than 3 minutes.
- Physiological monitoring alongside EEG monitoring could help to improve seizure detection.

## Introduction

Seizures commonly occur in neonates with conditions such as hypoxic-ischaemic encephalopathy and neonatal stroke.^1^ Prolonged uncontrolled seizures may constitute a neurologic emergency,^2^ and hence effective seizure detection is important. Signs of clinical seizure activity may be subtle in neonates and the relationship between clinical and electrographic seizures is poor with a large proportion of electrical seizures having no clinical manifestation.^3^

Continuous multichannel video-EEG is considered the gold standard for monitoring of seizures in neonates, however, the practical and interpretational challenges as well as resource implications of multichannel video-EEG limits its availability. Two-channel digital EEG monitoring alongside amplitude-integrated EEG (aEEG), is a practical and effective method for continuous neonatal EEG monitoring for the detection of seizures and assessing electrocortical background.^4–6^ Thus most centres in the UK rely on such monitors for continuous monitoring of neonates at risk of seizures,^7^ for example to monitor neonates receiving therapeutic hypothermia for hypoxic ischaemic encephalopathy (HIE).^8^

Seizures in preterm neonates may be associated with autonomic changes such as apnoeas,^9^ paroxysmal changes of heart rate,^10^ respiration and blood pressure.^11^ However, physiological changes such as oxygen saturation and respiratory patterns associated with seizures in term neonates have yet to be systematically studied. The aim of this study was to investigate whether electrographic seizures in term neonates are associated with physiological changes in respiratory patterns and oxygen saturation levels.

## Patients and Methods

Neonates were enrolled into the prospective ADAPTS (Automated Detection And Prediction of Term newborn Seizures) observational study on the neonatal unit at the Royal London Hospital, London. Term neonates admitted to the NICU, who had aEEG monitoring, as directed by the clinicians in charge of the clinical care, were recruited to study.

### Seizures

Two experienced neonatologists (DKS and ACK), who were blinded to the clinical information, identified unequivocal seizures independently with the aim of reaching a consensus when there was disagreement; a combination of clinical markers (for guidance) and the aEEG trace with confirmation from the raw EEG waveform were used to identify seizures. EEG seizure activity was defined as rhythmic spike and/wave activity lasting at least 10 seconds with evolution in seizure morphology and resolution.^12,13^ Impedance of the EEG electrodes was checked to be below 10kΩ.

### Data Acquisition

Left and right (C3, P3 and C4, P4) channels of EEG were monitored where clinically indicated using Olympic Brainz Monitor (OBM) cerebral function monitors (Natus Medical Incorporated, Middleton, Wisconsin). The two-channel EEG recordings were synchronised with a collection of high-resolution standard physiological measurements collected as part of routine neonatal intensive care. iCollect software (GE Medical Systems Ltd., UK) on a laptop PC connected to a GE Carescape B650 monitor (GE Medical Systems Ltd., UK) was used to acquire physiological data which were being monitored for clinical management. The physiological data recorded included, ECG, heart rate (HR), oxygen saturations (SpO_2_), respiratory rate (RR) and invasive blood pressure where monitored for clinical management. Oxygen saturations were measured using Masimo SET (signal extraction technology) probes (Masimo Corporation, Irvine, California) and modules in the Carescape monitor.

iCollect was used to collect ECG, oximetry plethysmography, respiration and invasive blood pressure waveforms with a sampling frequency of 300 Hz. In addition, trend data were acquired for SpO_2_, heart rate (from oximetry and ECG), respiratory rate and systolic, diastolic and mean blood pressure; were collected at a minimum rate of once per minute.

### Data analysis

Data were analysed using iCollect, Olympic Brainz Monitor Viewer, Excel (Microsoft Corporation, Redmond, Washington) and software we developed in MATLAB (The MathWorks Inc., Natick, Massachusetts); graphs were plotted using MATLAB and Minitab v19 (Minitab LLC, State College, Pennsylvania). Sections of traces with evidence of clear EEG seizure activity were compared with physiological data recorded at the same time. In this study we examined whether drops in SpO_2_ of at least 5% were associated with EEG seizures; the choice of 5% was from consideration of expected normal variability in SpO_2_. The trend graphs were compared with waveform displays to check the integrity of the data, for example that there was little or no artefact on the oximeter plethysmogram (pleth) waveform when assessing SpO_2_. The pulse rate from the SpO_2_ oximeter sensor (PR-SpO_2_) was checked to have no unexplained drops and to be consistent with the heart rate from ECG (HR-ECG) as an added procedure to avoid possible oximetry artefact.

## Results

Between June 2021 and June 2023–51 outborn and inborn term neonates admitted to the tertiary neonatal intensive care unit at the Royal London Hospital had two channels of EEG/aEEG monitored for at least 24 hours. Forty-four neonates were recruited to the study with informed consent of whom 22 babies had EEG-confirmed seizures. There was complete agreement between the two neonatal consultants as to which infants were having unequivocal EEG seizures; furthermore, there was agreement about the presence of EEG seizures for each of the individual episodes included in the following analysis.

Seventeen of the twenty-two term-born neonates had electrographic seizure activity observed together with simultaneous physiological measurements recordings. Of these recordings, 5 had technical issues such as artefact on the oximetry pleth trace at the time of the EEG seizures; although SpO_2_ values were mostly still recorded when there was artefact on the pleth trace, these data were not included in case the SpO_2_ reading might have been affected by the artefact. In addition, we did not include analysis in one infant who had repeated seizures with little clear break between them and for whom thus any physiological changes associated with seizures would be difficult to ascertain. Thus there were eleven neonates included in the analysis who had EEG seizures with simultaneous physiological data recorded in the first ten days after birth. The minimum recording period of EEG together with physiological monitoring was 5.5 hours with most recordings more than 24 hours long. Perinatal demographic details as well as clinical parameters, diagnoses and outcomes for these eleven neonates are shown in Tables 1 and 2 respectively.

**Table 1:** Perinatal characteristics of the 11 seizure neonates with physiological download analysis.

Perinatal characteristics – 11 term neonates	median (range) where applicable
Sex: Male *n*	8
Female *n*	3
Birth weight in grams	3315 (2370–3815)
Gestation (weeks)	38 (36–41)
Apgar score at 1 min	3 (0–9)
5 min	3 (0–8)
10 min	5 (0–9)
First pH	7.17 (6.8–7.49)
Lactate (mmol / L)	6.3 (0.8–26)
Seizures identified on aEEG	11/11
Clinical seizures	10/11
Anticonvulsant medications n	11/11
Ventilated *n*	10/11
HIE diagnosis *n*	7/11
Therapeutic hypothermia	7/11

**Table 2:** Clinical characteristics, diagnoses, outcomes of neonates with seizures.

Baby	Gestation (weeks)	Respiratory status at time of seizure		Seizure burden	Clinical seizure semiology	Laterality	Anti seizure medication used*	Muscle paralysis	Diagnosis(**)	MRI finding (***)	Outcome and neurological exam at discharge
		ventilated	FiO_2_								
1	38	Yes	100%	High	No clinical seizures	Right	Ph,Md	no	HIE	Acute ischemic change within the cortex of both cerebral hemispheres	Alive, normal
2	38	Yes	100%	High	Left arm twitching	Bilateral	Ph, Py, Md	yes	HIE	Not done	Died
3	40	Yes	21%	High	Left arm and leg clonic jerking	Bilateral	Ph, Py, Md	no	Perinatal Stroke	Bilateral ischemic changes of the left MCA territory with ischemic change within the left PLIC	Alive, central hypotonia with significant head lag
4	41	Yes	21%	Low	Rhythmic jerking of all four limbs	Bilateral	Ph	no	HIE	Normal	Alive, normal
5	38	Yes	100%	High	Extensor posturing of right arm and cycling movements	Bilateral	Ph	no	HIE	Severe hypoxic ischemic injury of bilateral basal ganglia, thalami and PLIC	Alive, tube feeds
6	39	Yes	21%	High	Left arm twitching with desaturations	Right	Ph	no	HIE	Normal	Alive, normal
7	36	Yes	21%	Low	Clonic jerks in arms	Bilateral	Ph, Md	no	HIE	Severe hypoxic ischemic injury of bilateral basal ganglia, thalami, PLIC, optic tracts	Alive, central hypotonia with significant head lag
8	40	No	21%	High	Left sided arm and leg → generalisation	Bilateral	Ph,Py,Md	no	IVH	Moderate volume intraventricular hemorrhage with early hydrocephalus	Alive, normal
9	41	Yes	100%	High	Tonic jerks left upper limb	Right	Ph. Md	yes	Perinatal Stroke	Encephalomalacia - distribution suggestive of right MCA infarction	Alive, tone abnormalities at discharge
10	37	Yes	100%	High	All four limbs - rhythmic jerking	Bilateral	Ph,Py,Md,Lt	yes	ARPKD	Severe global ischemic changes	Died
11	38	Yes	30%	High	All four limbs - rhythmic twitching and abnormal posturing	Bilateral	Ph, Md, Py	no	HIE, hypoglycemic	Severe white matter changes bilaterally	Alive, abnormal tone and movements at discharge and needed NG feeds

*Ph Phenobarbitone, *Py* Phenytoin, *Md* Midazolam, *Lt* Levetiracetam; Diagnosis column abbreviations (**): *HIE* hypoxic ischaemic encephalopathy, *ARPKD* autosomal recessive polycystic kidney disease. *MRI* column abbreviations (***): *MCA* middle cerebral artery, *PLIC* posterior limb of the internal capsule. Outcome abbreviation: *NG* naso-gastric.

### Physiologic monitoring

**Fig. 1:**
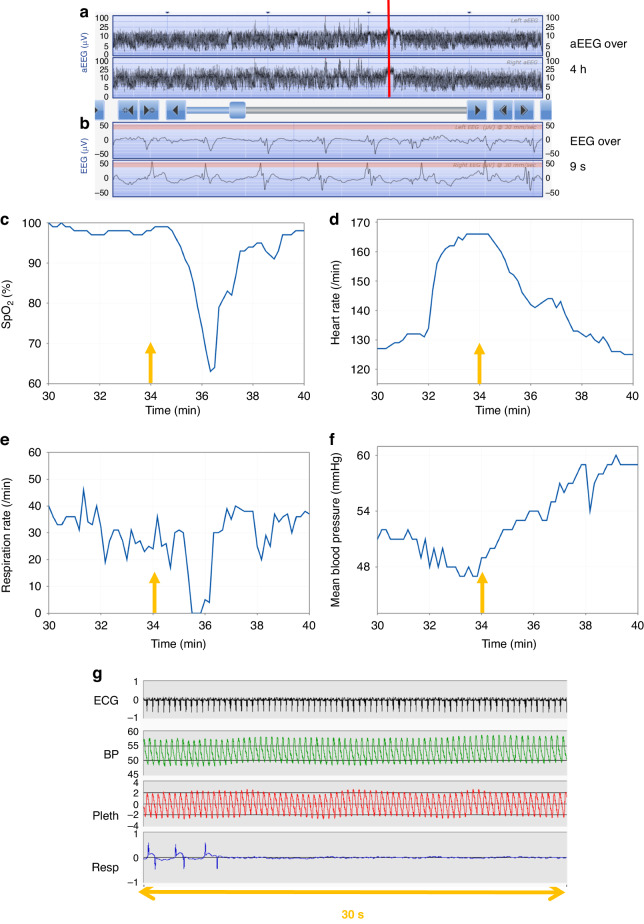
Example of EEG seizure episode associated with an apnoea, increase in heart rate and fall in oxygen saturation (SpO_2_) in a term ventilated neonate. The upper panel **a** shows the left and below that right aEEG trace over a four hour period with an increase in amplitude (red vertical line) for about 3 minutes which corresponds to seizure activity on the left and right raw EEG traces (panel **b**). The EEG seizure activity (orange arrows) was associated with a fall in oxygen saturation (SpO_2_) (panel **c**) and a drop in respiratory rate and short apnoea (panel **e**) shortly after the arrow time as well as an increase in heart rate (panel **d**) and increase in mean arterial blood pressure (panel **f**); these traces are shown over a period of ten minutes. A short 30 second period of corresponding ECG, blood pressure, oximetry plethymograph (pleth) and respiration trace at the start of the apnoea is shown in panel **g**; there is no suggestion of artefact on either the oximetry pleth or blood pressure trace.

**Fig. 2:**
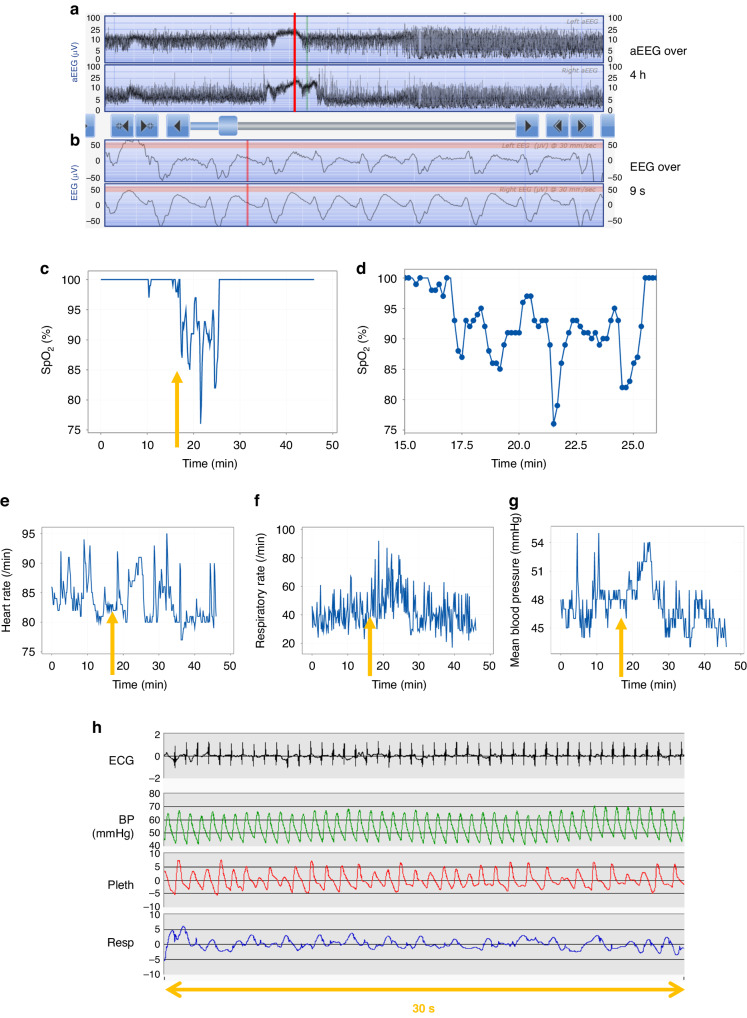
Example of a longer EEG seizure episode that is not associated with an apnoea but is associated with a fall in oxygen saturation (SpO_2_) and an increase in mean arterial blood pressure in a ventilated neonate. Panel **a** shows the left and below that right aEEG trace over a four hour period. There is an increase in overall amplitude of the aEEG (red vertical line) over about 15 minutes which corresponds to electrographic seizure on the raw EEG trace (panel **b**). The start of the EEG seizure activity, indicated with orange arrows, was associated with a fall in blood oxygen saturation (SpO_2_) as seen in **c** although there is some fluctuation in the values; a close-up of the SpO_2_ changes over 10 minutes is shown in **d** with blue dots for each individual SpO_2_ value. The heart rate (**e**) appears to increase although there is some fluctuation in earlier values and no drop in respiratory rate (**f**) shortly after the arrow time however, there is an increase in mean arterial blood pressure (**g**); traces **c**, **e**, **f** and **g** are shown over a period of fifty minutes in view of the length of the seizure activity. A short 30 second period of corresponding ECG, blood pressure, oximetry plethysmogram (pleth) and respiration trace corresponding to the seizure is shown in **h** in which the respiratory pattern appears variable.

In ten of the eleven neonates with seizures, at least one EEG seizure episode was associated with a fall in SpO_2_ of at least 5% and for nine of whom this decrease occurred over 3 minutes or less; 3 of these babies had evidence of apnoea (≥10 s) from impedance respiration monitoring. An example of a seizure associated with a decrease in SpO_2_, apnoea as well as an increase in heart rate (HR) is shown in Fig. 1. This example shows an unclear increase in aEEG amplitude associated with a clear seizure on the corresponding raw EEG of 3 minutes duration which could thus potentially be missed. Figure 2 shows an example of a longer duration seizure (15 minutes) accompanied by left arm twitching and associated with a drop and some fluctuation in SpO_2_ over about ten minutes; in this example there was no accompanying apnoea although there is some artefact on the respiratory trace.

**Fig. 3:**
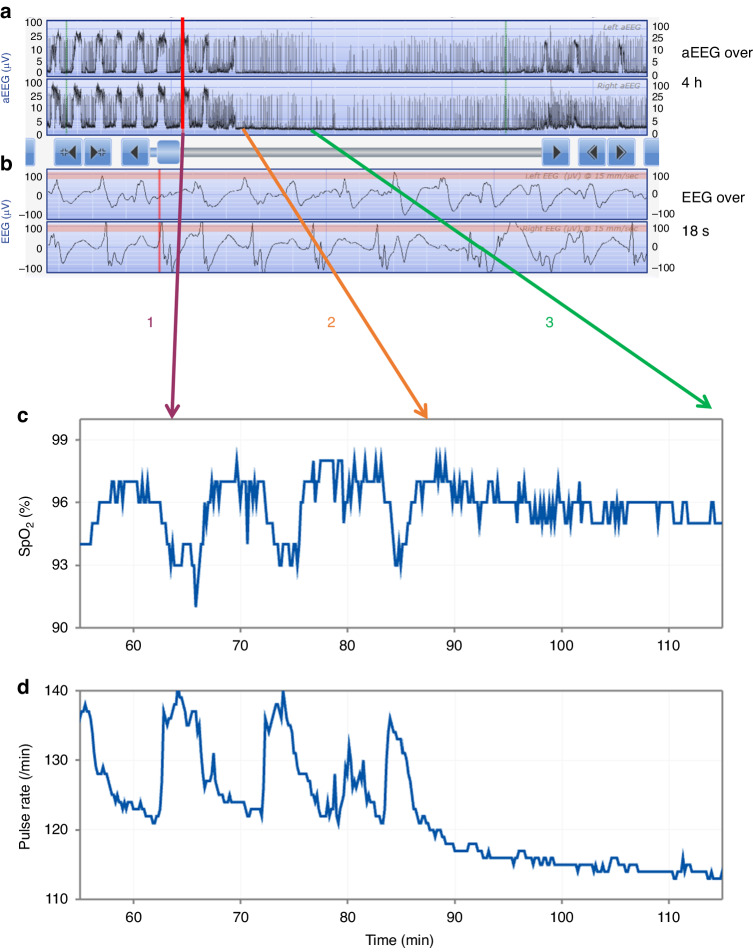
Example of repeated EEG seizure episodes associated with increases in heart rate and fall in oxygen saturation (SpO_2_) in a ventilated neonate receiving muscle paralysis. The upper panel **a** shows the left and below that right aEEG trace over a four hour period with an increase in amplitude as in the example with the red vertical line which corresponds to seizure activity on the left and right raw EEG traces (panel **b**). The EEG seizures were associated with a fall in oxygen saturation (SpO_2_) (panel **c**) and example purple line (1) and an increase in pulse rate (panel **d**) which stop after the end of the seizure activity example lines 2 (orange) to 3 (green); these traces are shown over a period of sixty minutes.

Figure 3 shows an example of repeated seizures with relatively short inter-ictal periods in an infant receiving muscle paralysis for ventilation. Associated with each seizure episode was a decrease in SpO_2_ which was preceded by an increase in pulse rate measured by the oximeter. In the period shown after seizures there is no clear change in heart rate or SpO_2_.

**Fig. 4:**
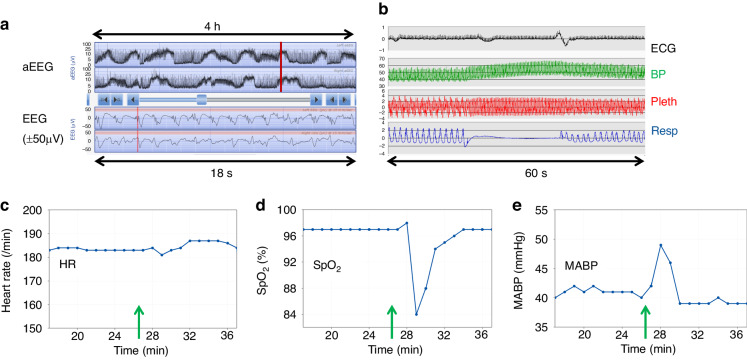
Example of EEG seizures (A) in a ventilated infant receiving muscle paralysis. The upper panel in **a** shows the left and below that right aEEG trace over a four-hour period with an increase in amplitude as in the example with the red vertical line which corresponds to seizure activity on the left and right raw EEG traces plotted over 18 seconds below the aEEG. The raw ECG, blood pressure, oximetry pleth and respiratory waveforms are shown over a one-minute period in **b**; although the trace is compressed over 60 seconds, there is no suggestion of artefact on either the oximetry pleth trace or the blood pressure trace. The lower graphs in **c**, **d** and **e** show the heart rate (HR), SpO_2_, and mean arterial blood pressure (MABP) over 18 minutes just before and after the seizure start indicated by the green vertical arrow. In addition to the acute decrease in SpO_2_ (**d**), a preceding apnoea can be seen on the respiratory trace (**b**) as well as change in the pleth pattern from one with respiratory modulation to no clear respiratory modulation in the apnoeic period (**b**); similar changes are seen in the blood pressure trace pattern (**b**). There is a baseline tachycardia and no clear change in heart rate seen associated with the seizure episode (**c**) however, there is an increase in mean arterial blood pressure associated with the seizure (**e**).

Taking one measurement for each of the ten neonates who had a decrease in SpO_2_ associated with seizures, the median (range) fall in SpO_2_ was 13.5 (5 to 35)% and increase in HR was 7.5 (0 to 30) /min; six of the ten infants had invasive blood pressure recording and for mean arterial blood pressure (MABP) a median (range) increase of 6.5 (0 to 13) mmHg was seen associated with EEG seizures. One neonate had a background tachycardia (baseline HR 180 /min) and no rise in heart rate was noted associated with an EEG seizure although that infant had an SpO_2_ decrease of 13% and increase in MABP of 8 mmHg (Fig. 4). This infant had suffered severe multiorgan failure, was on multiple inotropes and subsequently died.

Three infants with falls in SpO_2_ associated with seizures were receiving muscle paralysis medication as part of the ventilation support; two of these babies showed clear evidence of apnoea on the respiratory trace prior to the fall in SpO_2_ even though they were on synchronized intermittent mandatory ventilation (SIMV) ventilation with 100% fraction of inspired oxygen (FiO_2_) as seen in the example in Fig. 4. The evidence of apnoea was supported by a change in pleth signal pattern during the apnoea in which the respiratory modulation of the pleth and invasive blood pressure was absent as seen in Fig. 4; following the apnoea, respiratory modulation can again be seen on both the oximetry pleth and BP traces albeit less than before which is consistent with lower amplitude excursions on the impedance respiratory trace. The baby had a heart rate of about 180/minute and no clear change in heart rate was seen associated with the seizure episode (C); this may suggest that there was little capacity to further increase heart rate in view of the baseline tachycardia.

Nine of the ten neonates with drops in saturations were mechanically ventilated, 6 were in supplementary oxygen; all nine ventilated infants received morphine infusion for sedation.

In order to assess how often acute decreases in SpO_2_ can be associated with seizure activity, we reviewed the physiological and EEG recordings over a period of 5.5 to 12 hours (median 12 hours) in the ten neonates with oxygen saturation drops. The criteria for identifying an acute fall in SpO_2_ were that there was an SpO_2_ decrease of at least 5% occurring within 3 minutes and with little or no artefact on the oximetry pleth trace; the decreases were followed by a subsequent increase in SpO_2_ as seen in Figs. 1 to 4. Table 3 summarises the number of SpO_2_ falls of at least 5% together with the number associated with EEG seizures and for those the minimum and maximum drop of at least 5% in SpO_2_. The median (range) number of acute SpO_2_ falls was 7 (2 to 20) of which there were seizures in 3 (1 to 20) episodes in the review period; the median (range) percentage episodes of SpO_2_ falls that were associated with seizures was 50 (16 to 100) % in that period.

**Table 3:** Details about decreases in SpO_2_ of at least 5% and those associated with EEG seizures as well as apnoeas associated with SpO_2_ decreases and seizures in the duration of recording examined; the total number EEG seizures in the examined period is also shown.

Baby	Number of SpO_2_ drops ≥ 5%	Duration (hours)	Number of EEG seizures in review period	Number of SpO_2_ drops ≥ 5% with seizures	Minimum SpO_2_ drop with seizures (%)	Median SpO_2_ drop with seizures (%)	Maximum SpO_2_ drop with seizures (%)
1	0	12	10	0	–	-	-
2	6	12	13	3 (2 apnoeas)	8	8	13
3	19	12	18	3 (2 apnoeas)	11	20	30
4	10	11	3	2	6	17	28
5	5	8	14	4	10	12	16
6	5	12	2	2	15	18	20
7	2	12	1	1	8	8	8
8	20	6	48*	20	5	6	15
9	2	5.5	19	2 (2 apnoeas)	5	8	11
10	15	12	18	14	5	5	6
11	7	12	10	2	5	7	8

*the EEG for this baby indicated status epilepticus.

## Discussion

We objectively demonstrate that acute drops in oxygen saturation are not uncommon in association with seizures in sick term neonates at risk of seizures and undergoing intensive care, with a drop of at least 5% oxygen saturation noted in at least one seizure in ten of eleven neonates studied; in three neonates, there were SpO_2_ decreases with seizures that were also associated with apnoeas as shown in Table 3. The desaturations were also noted in neonates receiving mechanical ventilation and supplementary oxygen. In addition, for the 10 babies who had falls in SpO_2_ associated with the EEG seizure activity, an increase in heart rate was also noted in 8 of the 10 babies and an increase in mean arterial blood pressure in 4 of 6 babies. The median (range) percentage episodes of SpO_2_ decreases of at least 5% that were associated with seizures was 50 (16 to 100) % in the reviewed period.

Although anecdotally reported,^14^ we are not aware of previous studies investigating changes in oxygen saturation and respiratory rate in term neonates with electrographic seizure activity in term infants. A recent study in preterm infants,^15^ also observed that some infants had falls in SpO_2_ associated with seizures; in that study oximetry pleth trace recording was not reported, however, we used the pleth to check for artefact that could potentially affect the indicated oxygen saturation. Apnoea, as the sole clinical manifestation associated with a seizure, has been described in neonates,^16^ and may be characteristic of focal epilepsies localised to the temporal lobe and associated structures.^17^ Ictal apnoea may be the only clinical manifestation in temporal lobe epilepsies and the apnoea may precede EEG changes and the onset of the clinical seizures in children.^18^ Ictal apnoeas have also been described in term neonates with neonatal temporal lobe haemorrhages.^19^

Plausible mechanisms of autonomic responses during seizures may be explained by changes affecting the central autonomic network (CAN), comprising of the insula and the medial prefrontal cortex, amygdala, hypothalamus and parts of the brainstem,^20^ which may exert control over the sympathetic and parasympathetic, neuroendocrine, respiratory and sphincter motor neurons.

Oxygen desaturations have been reported in association with seizures in children with epilepsy associated with apnoea and hypoventilation.^21^ In adult patients with intractable epilepsy oxygen desaturation, often in association with hypopnea and apnoea, has been noted particularly in patients with right sided temporal lobe epilepsy.^22^ In patients of 16 years and over, prolonged apnoeas of 60 s and greater have been associated with severe hypoxaemia with oxygen saturation <75% and have been considered at risk of sudden unexpected death in epilepsy (SUDEP).^23^ Furthermore using Near-Infrared Spectroscopy falls in cerebral oxygenation associated with aEEG seizures have been observed in a 35 week gestation infant.^24^

Interestingly, three ventilated neonates with seizures showed concomitant apparent apnoeic events on respiratory monitoring prior to falls in SpO_2_ including two receiving muscle paralysis for ventilation. The change in respiratory pattern detected from the impedance respiratory trace was supported by interruption in respiratory modulation of the oximetry pleth trace,^25,26^ together with a similar effect seen on the invasive blood pressure trace. The reason why three infants could still show marked diminution or absence of respiratory effort on monitoring despite being mechanically ventilated is not clear to us but we speculate that this could be related to ‘splinting’.^27^ There was no indication of a mechanical ventilation equipment problem.

There is a paucity of data in neonates who have oxygen desaturations in association with seizures. Seizure burden has been shown to be associated with brain injury in neonates who have undergone therapeutic hypothermia after HIE,^8,28^ and neonatal seizure burden has been shown to be associated with adverse neurologic outcomes.^29,30^ Although the SpO_2_ level at the time of the drops associated with seizures was still greater than that expected just before birth,^31,32^ it is unclear whether additional hypoxemic events associated with seizures could contribute to adverse outcomes in these at-risk neonates.

Limitations of this study include that using aEEG with raw EEG may miss short and focal seizures.^30^ However this technique has been shown to have moderately good sensitivity and specificity for seizure detection.^5,33^ It is less resource intensive and is applied by the nursing and medical teams relatively quickly after the admission of the baby to the neonatal unit, in circumstances where the availability of conventional EEG may be more limited; hence it is considered a pragmatic approach of enabling continuous monitoring of neonatal cerebral function which is widely adopted in the UK.^7^

EEG and physiological data monitored as part of standard neonatal intensive care were successfully synchronised and recorded to enable traces to be compared easily. In our approach, not only summary trends in physiological data were recorded but also the waveforms allowing increased confidence in inclusion of good quality data and exclusion of artefacts. We suggest that taking account of unexplained acute drops in SpO_2_ could help improve detection of seizures in vulnerable term babies particularly if the seizures are of short duration and could potentially otherwise be missed.

## Conclusions

Our results indicate that term neonates with seizures not infrequently suffer from falls in oxygen saturation which may not be accompanied by an apnoeic episode; most of the infants in our study had either a diagnosis of HIE or perinatal stroke. We suggest that if unexplained decreases in oxygen saturations in at-risk term neonates are observed, seizures should be considered as possibly being part of the aetiology.

## Data Availability

Data are available upon reasonable request.
